# A new tool-using bird to crow about

**DOI:** 10.3758/s13420-017-0262-5

**Published:** 2017-03-31

**Authors:** Natalie Uomini, Gavin Hunt

**Affiliations:** 1 Max Planck Institute for the Science of Human History, Kahlaische Strasse 10, 07745 Jena, Germany; 2 238 Meola Road, Auckland, 1022 New Zealand

**Keywords:** Tool use, Comparative cognition, Corvid

## Abstract

The Hawaiian crow has been revealed as a skilled tool user, confirmed by testing the last members of this endangered species that survive in captivity. The finding suggests its behavior is tantalizingly similar to that of the famous tool-using New Caledonian crow and has implications for the evolution of tool use and intelligence in birds.

Tool use is often singled out as a defining feature of humanity. However, many other animals have independently evolved technological skills. They are phylogenetically widespread yet occur at low frequency within different animal groups. Why should some species evolve tool use but not others? Various preconditions are proposed: cognitive, environmental, anatomical, behavioral, and genetic. Primates and birds probably shared some key features that enabled their tool skills to evolve in parallel. Intelligence, behavioral flexibility, and innovation are just a few possibilities. Corvids, with relatively large brains and impressive cognitive abilities, are prime candidates to evolve technology. One such corvid is the New Caledonian crow (*Corvus moneduloides*), whose tool-making talents rival those of our close relatives (Hunt & Uomini, 2016).

Rutz et al. (2016) report that the Hawaiian crow (*Corvus hawaiiensis*) is also a tool-using species. Sadly, the Hawaiian crow is extinct in the wild. The world’s population is sustained in two breeding facilities in the hope they can eventually return to the wild. From previous work with New Caledonian crows, Rutz and colleagues predicted in 2011 that Hawaiian crows *should* be tool users (Rutz & St Clair, 2012). Meanwhile, the Hawaiian crow keepers had commonly observed tool use in their crows. They began collaborating in 2012. The fact that this publication was 4 years in the making testifies to the extensive research the team carried out to explore some of the key factors underpinning Hawaiian crow tool use. We focus here on innovation, genetic basis, and social transmission.

Rutz et al. (2016) tested captive Hawaiian crows with the same tool-use tasks as those given to New Caledonian crows: natural logs with meat in holes that could only be reached with suitably long tools. About 90% of adult crows and 40% of juveniles used twigs to successfully extract the food (81 of 104 birds). The birds were quick to pick up twigs and solve the task. The videos show that adult crows chose tools of appropriate length or shortened overly long twigs. However, unlike New Caledonian crows, the Hawaiian crows did not appear to standardize their tools, as they used plant materials of many sizes and shapes.

The Hawaiian crows were already familiar with tools, for example to fish food from their water bowls with sticks. Because tool use is known only in these captive birds, it is possible that the behavior was an innovation by one or more smart birds. Such innovation was documented in a crow relative that does not use tools in the wild, the rook (*Corvus frugilegus*), which spontaneously used tools to get food in captivity. However, that most adult Hawaiian crows successfully solved the log task suggested an inherited disposition for tool use, similar to that in the New Caledonian crow.

To find out if Hawaiian crows’ technological skills had a genetic basis, Rutz et al. (2016) reared seven juveniles deprived of social contact to other tool users (crow and human). These juveniles readily manipulated objects and tried to reach inaccessible food with them. However, they spent less time manipulating objects than New Caledonian crows and non-tool-using ravens (*Corvus corax*) of the same age did and did not show the stereotyped probing behaviors preceding functional tool use seen in New Caledonian crows. After the experimenters provided sticks and made the holes easier to access, three crows made 13 successful probes (out of 96 attempts in the new setup). Only one juvenile made repeated successful food extractions using tools by the end of the 5.5 month study period. These findings show that Hawaiian crows have a rudimentary genetic predisposition to use tools. Because New Caledonian crows take 1 to 2 years to reach adult proficiency with tools, it will be interesting to study the ontogeny of tool use in Hawaiian crows over a similar time period.

Although naïve juveniles are able to independently develop tool use, their mixed success at extracting food with tools left open the possibility that the widespread tool skills in the captive population were invented by clever birds and then spread by social learning. To test this directly, Rutz et al. (2016) did a nifty bit of social network modeling of long-term data in the crows’ housing group histories. The captive breeding program began in 1970. Using detailed records from 1996 to 2013, Rutz et al. (2016) determined which pairs of birds were in visual contact at any time and simulated the learning pathways leading from each current tool user back in time. Their computer model calculated how many crows would have had to innovate tool use over the years in order for all current 74 tool users (excluding the seven naïve juveniles) to have socially learned tool use. If learning occurs every time a juvenile observes an adult, eight independent innovation events would have been required. Thus, it was unlikely that tool use was repeatedly invented in the captive population and spread by social transmission alone. Even if the nine wild-caught crows who founded the original captive population had socially obtained tool skills in the wild, it is more parsimonious that the widespread behavior in the current population is underpinned by an inherited disposition specifically for tool use.

If Hawaiian crows indeed used tools in the wild, we have a window into their minds. Hunt, Gray, and Taylor (2013) proposed three cognitive capacities required for flexible tool use in birds. First, a bird needs to learn that an object can be used to solve a problem. Second, it must have the working memory to deal with the procedural complexity involved in tool use. Third, it must acquire the motor skills to manipulate a tool in a controlled way. The videos published by Rutz et al. (2016) suggest that adult Hawaiian crows clearly understand the problem (the food is in the hole) and the solution (use a stick to get the food out). However, the way Hawaiian crows treat their tools offers a possible clue to their working memory capacity. They do not appear to safeguard their tools. After each probe, they drop the tool before pecking at the food. In contrast, New Caledonian crows usually safeguard their tools in between probes by deliberately and carefully trapping them under a foot, on a branch, or in a hole. The difference could be related to working memory, motivation, or necessity (e.g., although New Caledonian crows retain their tools even near the ground, Hawaiian crows might safeguard their tools if using them high above ground). Although adult Hawaiian crows succeed at getting food with tools, they appear to lack the refined tool control with the bill that is a hallmark of New Caledonian crows. This might be due to their curved bill, contrasting with the New Caledonian crow’s straight bill which makes tool manipulation more efficient. In sum, even if a species understands the advantage of tool use, sufficient working memory and manipulation skill are probably important for long-term maintenance of the behavior.

It is always exciting when an animal species is shown to have tool-using abilities. The Hawaiian crow joins the 170 bird species that can use simple tools in captivity and/or in the wild. The species is clearly innovative with an inherited disposition for functional object manipulation. It will be difficult to show conclusively that the Hawaiian crow habitually made and used tools in the wild, or evolved any technological traditions. We hope ongoing research will investigate aspects such as life history, social learning, and foraging behavior to help answer these questions. The Rutz et al. (2016) study opens up exciting prospects for future comparative work on the evolution of avian tool use, for instance, on bill morphology, binocular vision, manipulation precision, cognitive abilities, brain size, and genetic underpinnings. It might also help answer the question of why avian tool use is more likely to evolve on tropical islands. Is it because of reduced competition for embedded prey and low predation, as the authors suggest, or extreme seasonality in the abundance of nonembedded prey that motivates a clever bird to use tools to get embedded prey in hard times?
